# Supplementary figures and images for: 3-Dimensional organization and dynamics of the microsporidian polar tube invasion machinery
Source: PLoS Pathog. 2020 Sep 18;16(9):e1008738. doi: 10.1371/journal.ppat.1008738 (PMC7526891; doi:10.1371/journal.ppat.1008738)

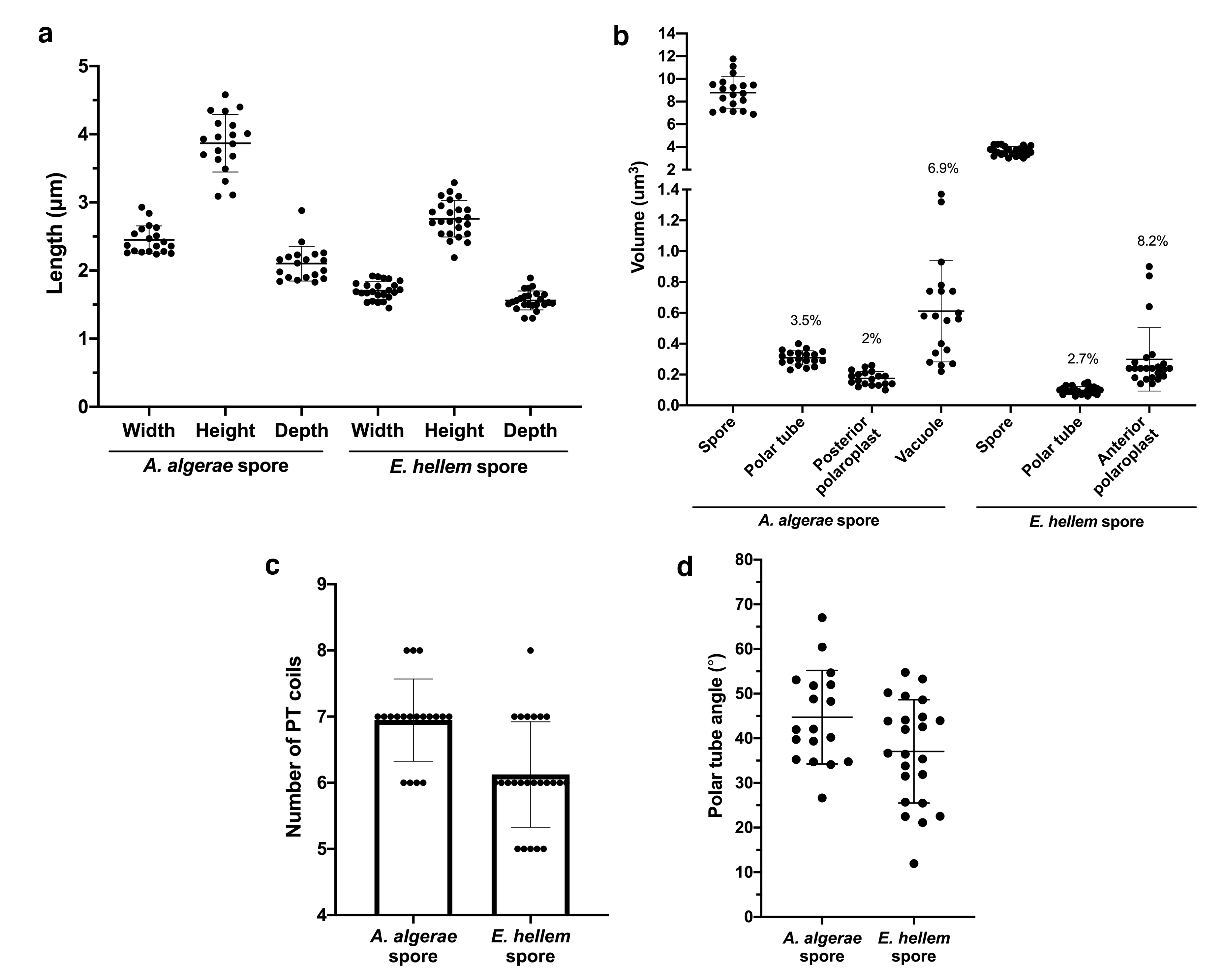

Supplement: S1 Fig — (a) Quantification of spore dimensions. (b) Quantification of volumes. Organelle volumes as a percentage of the entire spore volume are noted on the graph. (c) Number of PT coils quantified from 3D reconstruction of spores. (d) Quantification of the angle between PT coils and the A-P axis. All error bars in this figure represent standard deviation (n = 19 for A. algerae and n = 23 for E. hellem). (TIF) [file ppat.1008738.s001.tif]

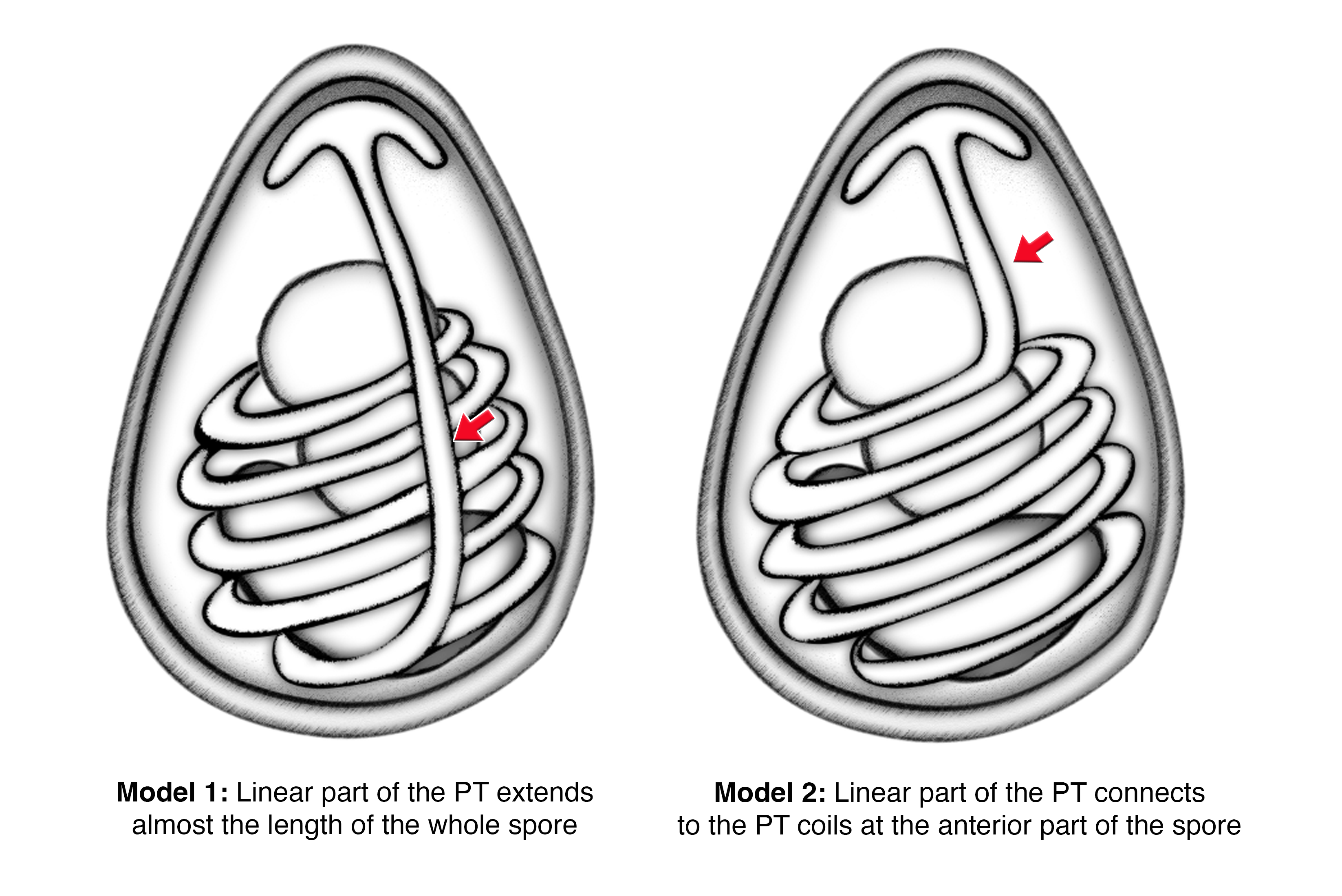

Supplement: S2 Fig — Schematic diagrams showing two possible models of the connection between straight and coiled regions of the PT. Red arrows indicate the region where the PT is straight. Model 1 was proposed by Cali et al[46]. (TIF) [file ppat.1008738.s002.tif]

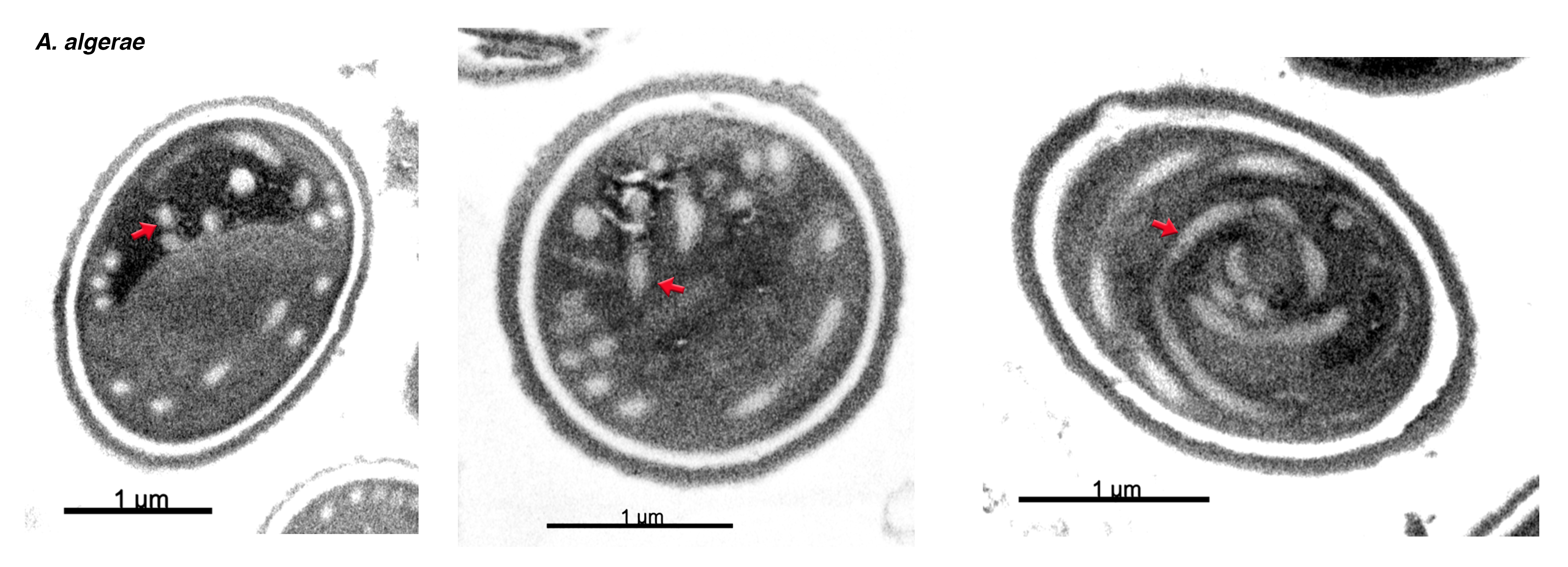

Supplement: S3 Fig — Three representative SBFSEM sections originating from spores with tangled PT ends, as described in Fig 2D. Red arrows indicate the PT. (TIF) [file ppat.1008738.s003.tif]

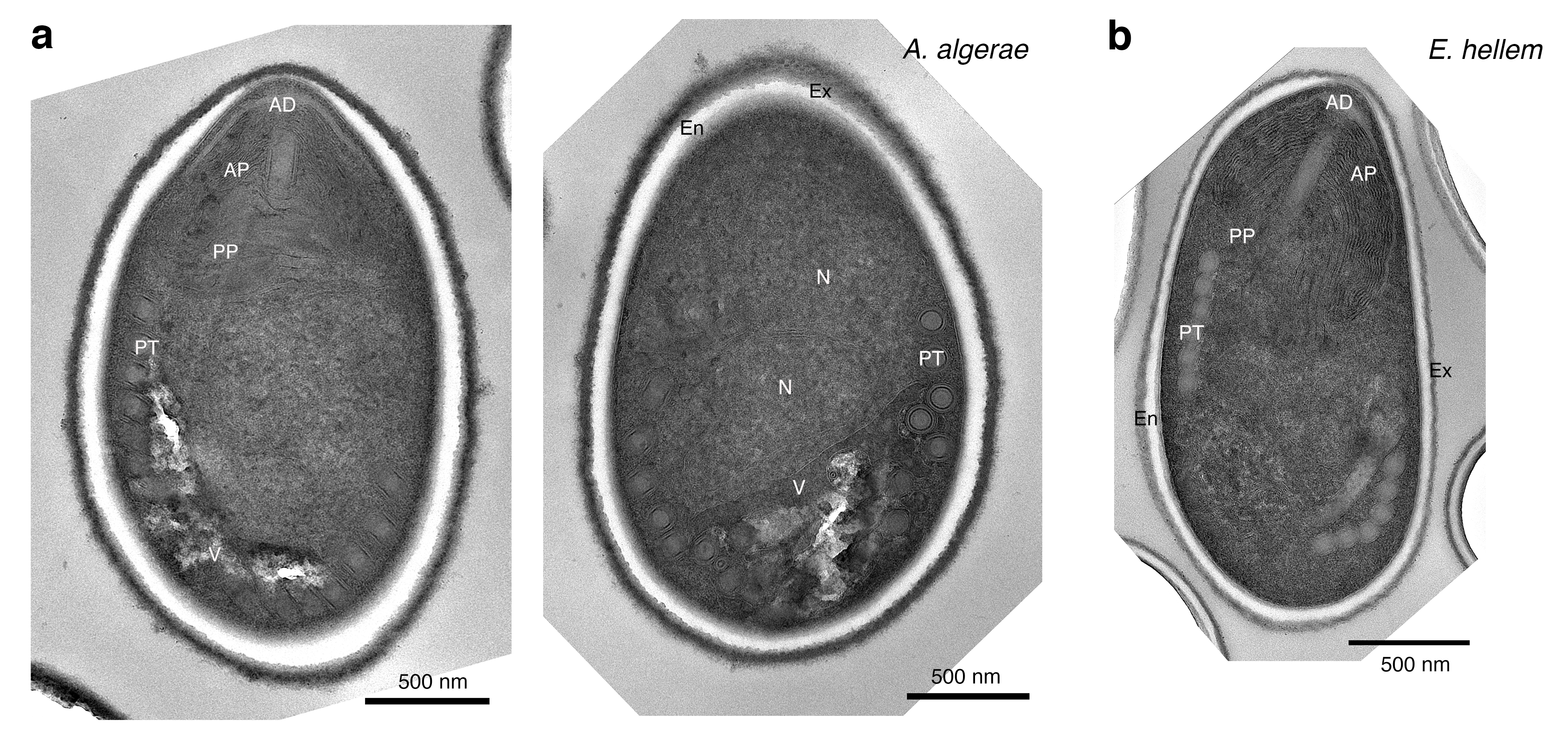

Supplement: S4 Fig — TEM sections of (a) A. algerae spores and (b) E. hellem spore indicating structures inside the spore, including exospore (Ex), endospore (En), anchoring disc (AD), anterior polaroplast (AP), posterior polaroplast (PP), polar tube (PT), nucleus (N), and vacuole (V). These samples were used for SBFSEM experiments. The right panel of (a) is the same as that shown in Fig 2E, but without color overlay. (TIF) [file ppat.1008738.s004.tif]

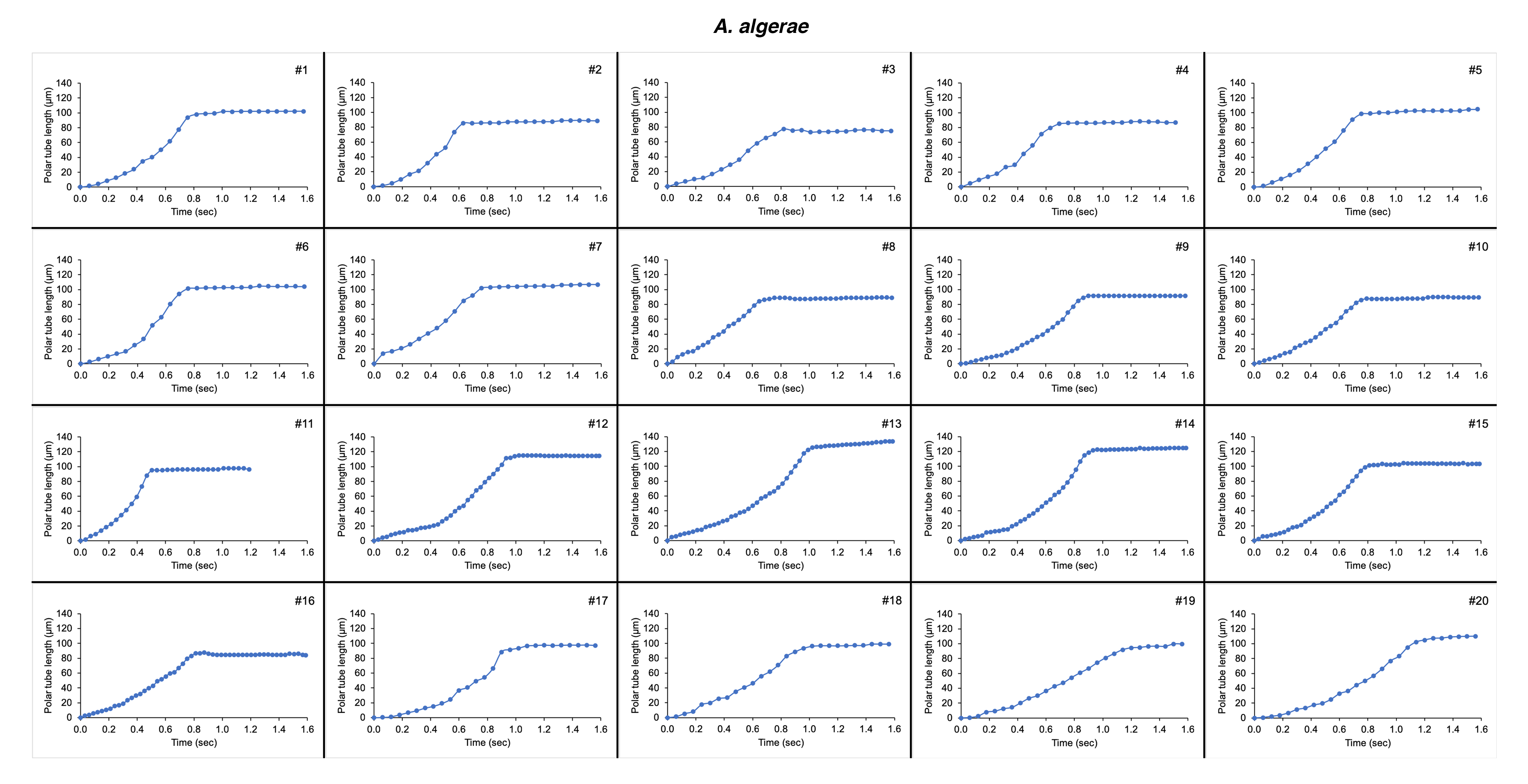

Supplement: S5 Fig — Graphs represent polar tube length over the time period of PT germination for 20 individual spores. See S2 Table for data used to generate these plots. (TIF) [file ppat.1008738.s005.tif]

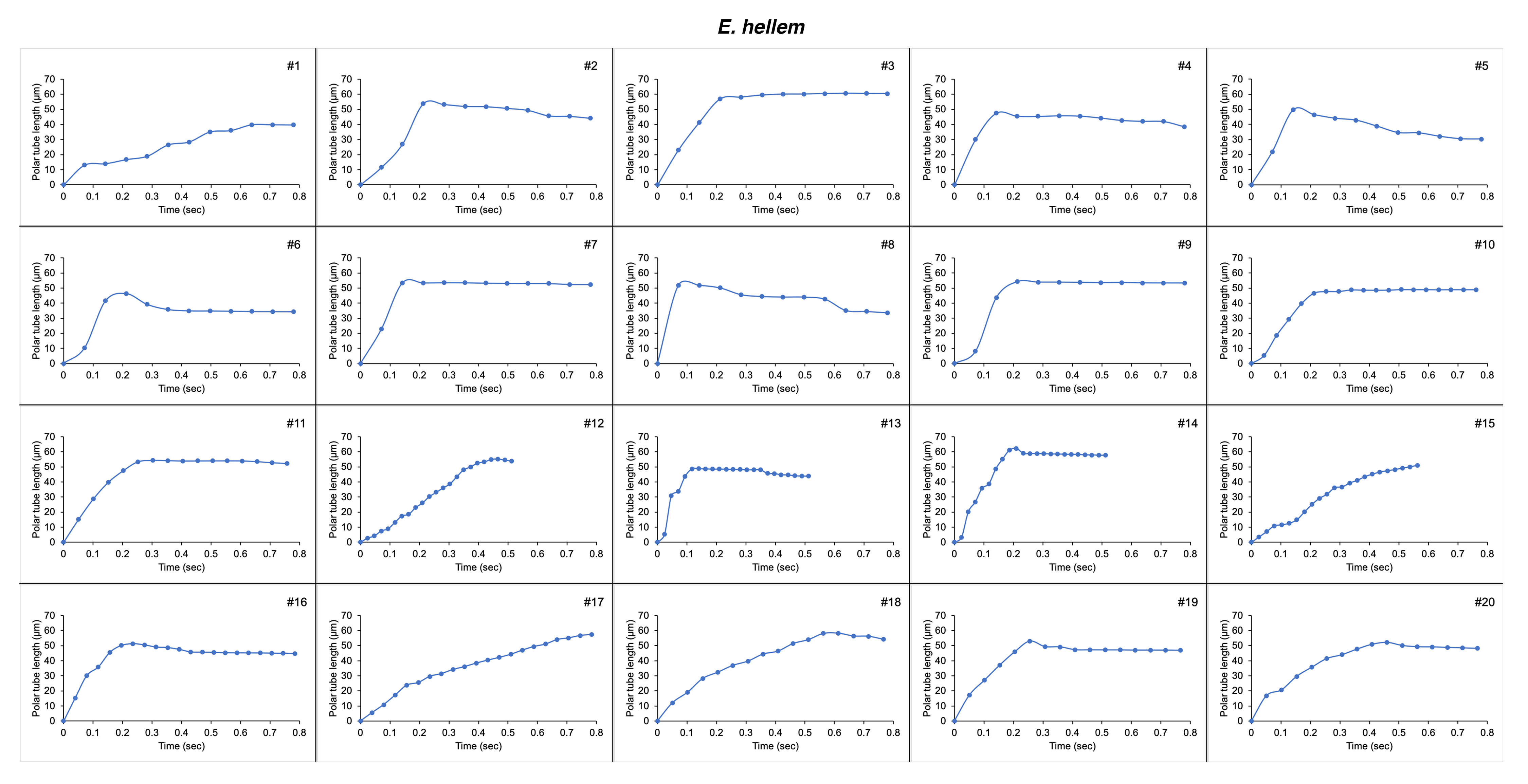

Supplement: S6 Fig — Graphs represent polar tube length over the time period of PT germination for 20 individual spores. See S2 Table for data used to generate these plots. (TIF) [file ppat.1008738.s006.tif]

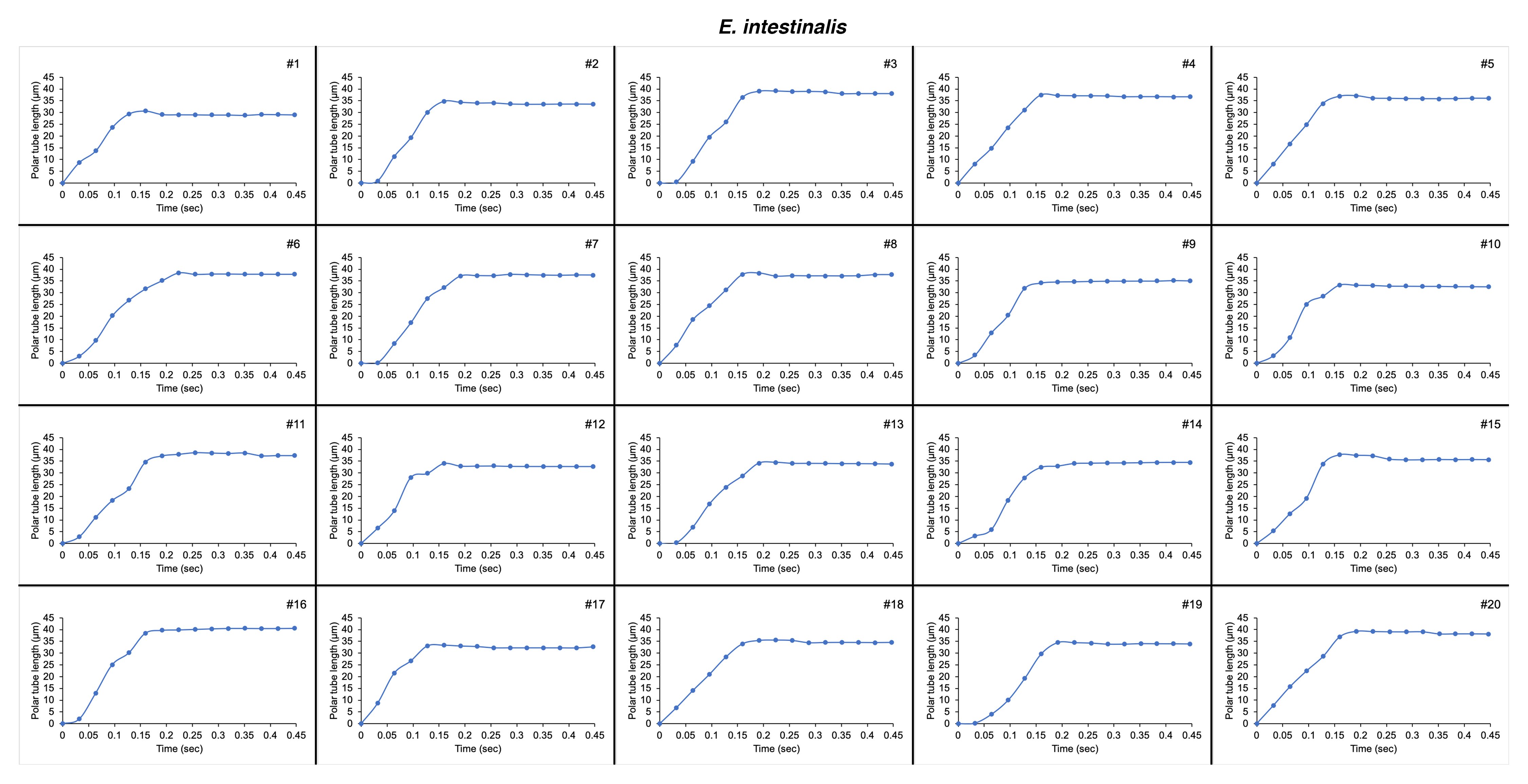

Supplement: S7 Fig — Graphs represent polar tube length over the time period of PT germination from 20 individual spores. See S2 Table for data used to generate these plots. (TIF) [file ppat.1008738.s007.tif]

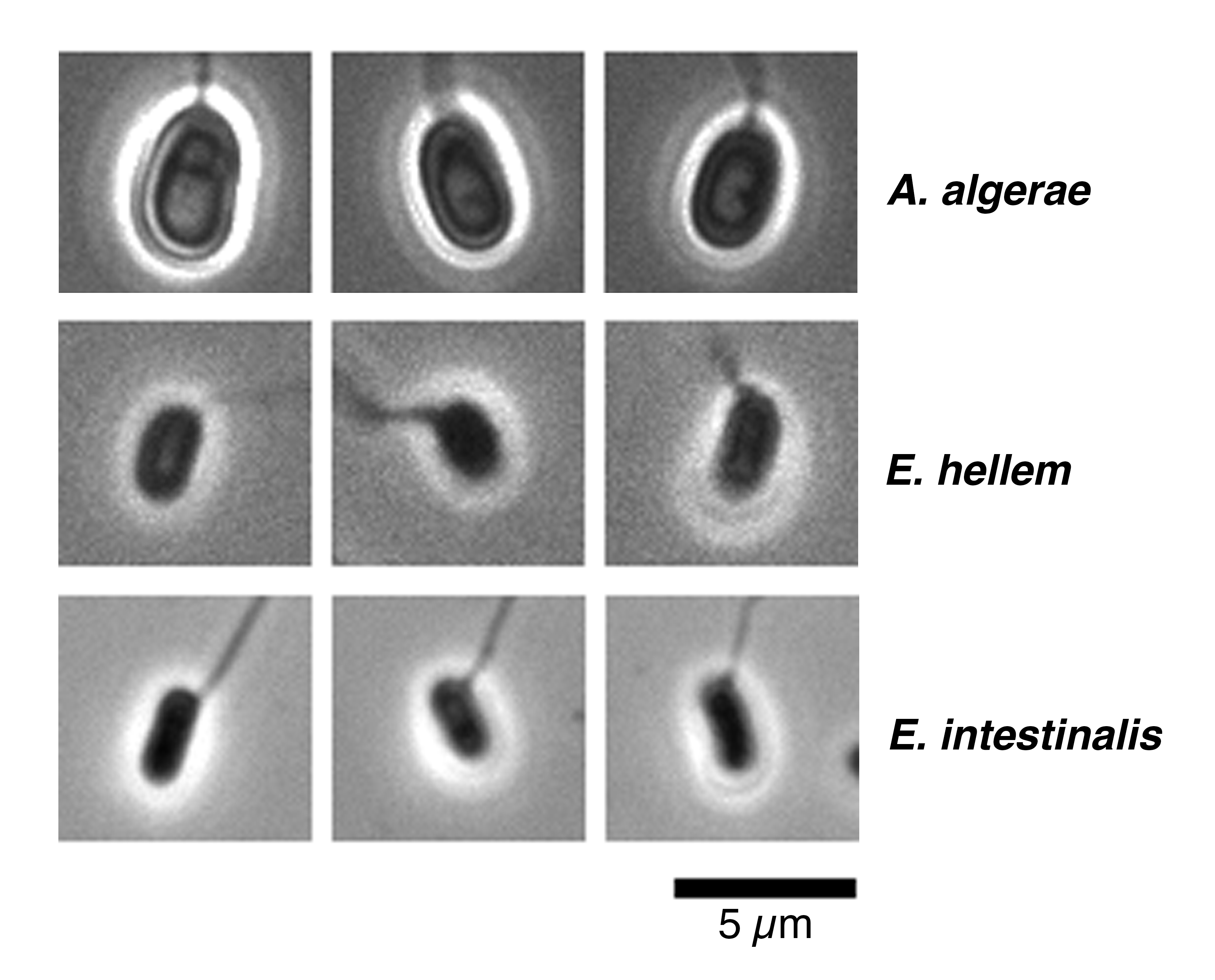

Supplement: S8 Fig — For A. algerae, 100% of the PTs emerge from the center (apical tip) of the spore, while for E. hellem and E. intestinalis 69% and 88% of PTs emerge off-center relative to the apical tip, respectively. (TIF) [file ppat.1008738.s008.tif]

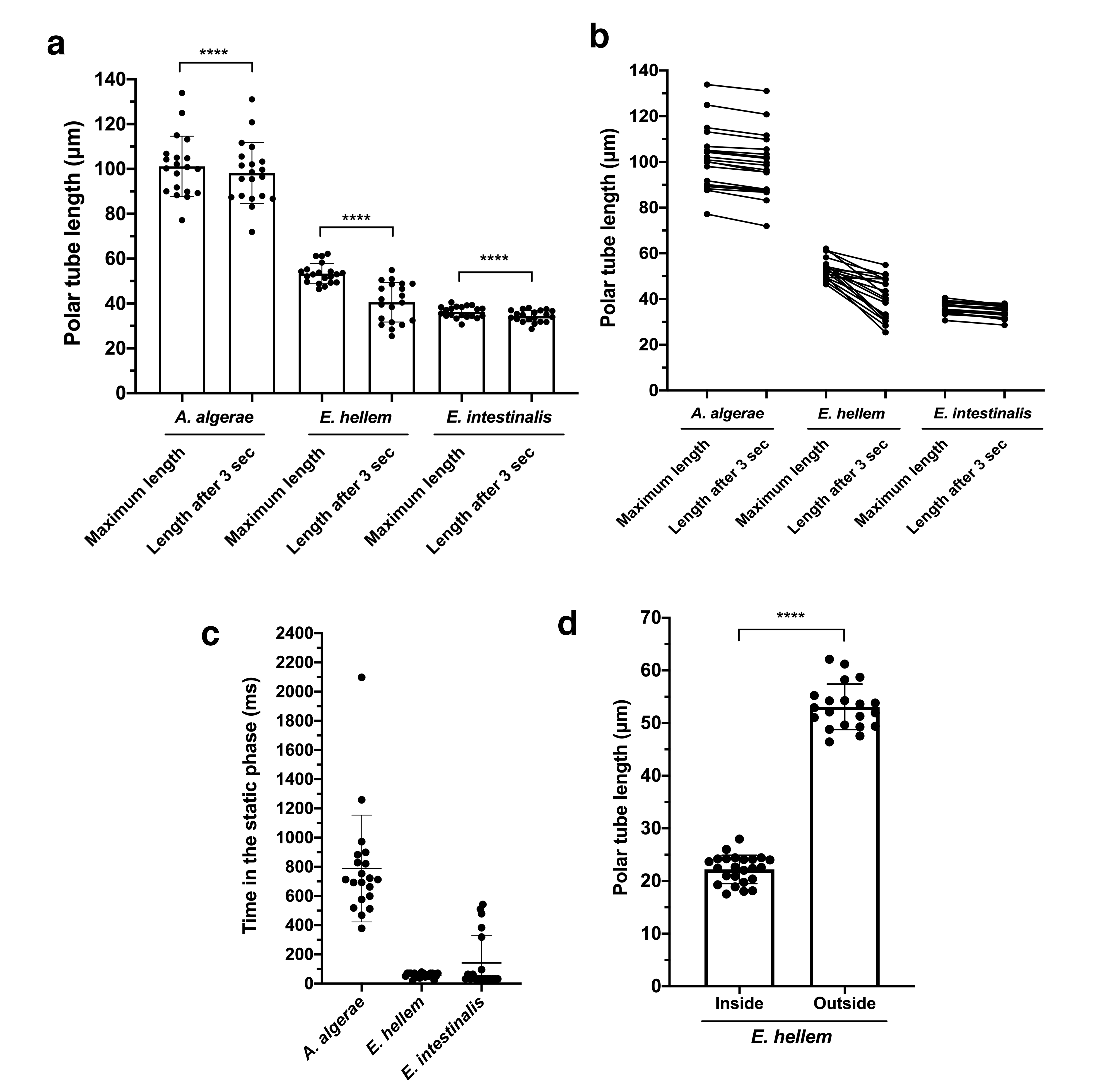

Supplement: S9 Fig — (a) Graph showing the maximum length and the length of the PT at 3 sec after germination is complete, ****p<0.0001 (paired Student’s t-test). (b) Same data as in (a). Each line connects the PT from the same spore, before and after shortening. (c) Time spent in phase II of germination, when the PT is static. (d) Quantification of PT length in the intact spores from SBFSEM and the length of fully extended PT from optical microscopy. The graph is the same as presented in Fig 4H, but with individual data points plotted. ****p<0.0001 (unpaired Student’s t-test). Error bars in this figure represent standard deviations (n = 20 for each species). (TIF) [file ppat.1008738.s009.tif]

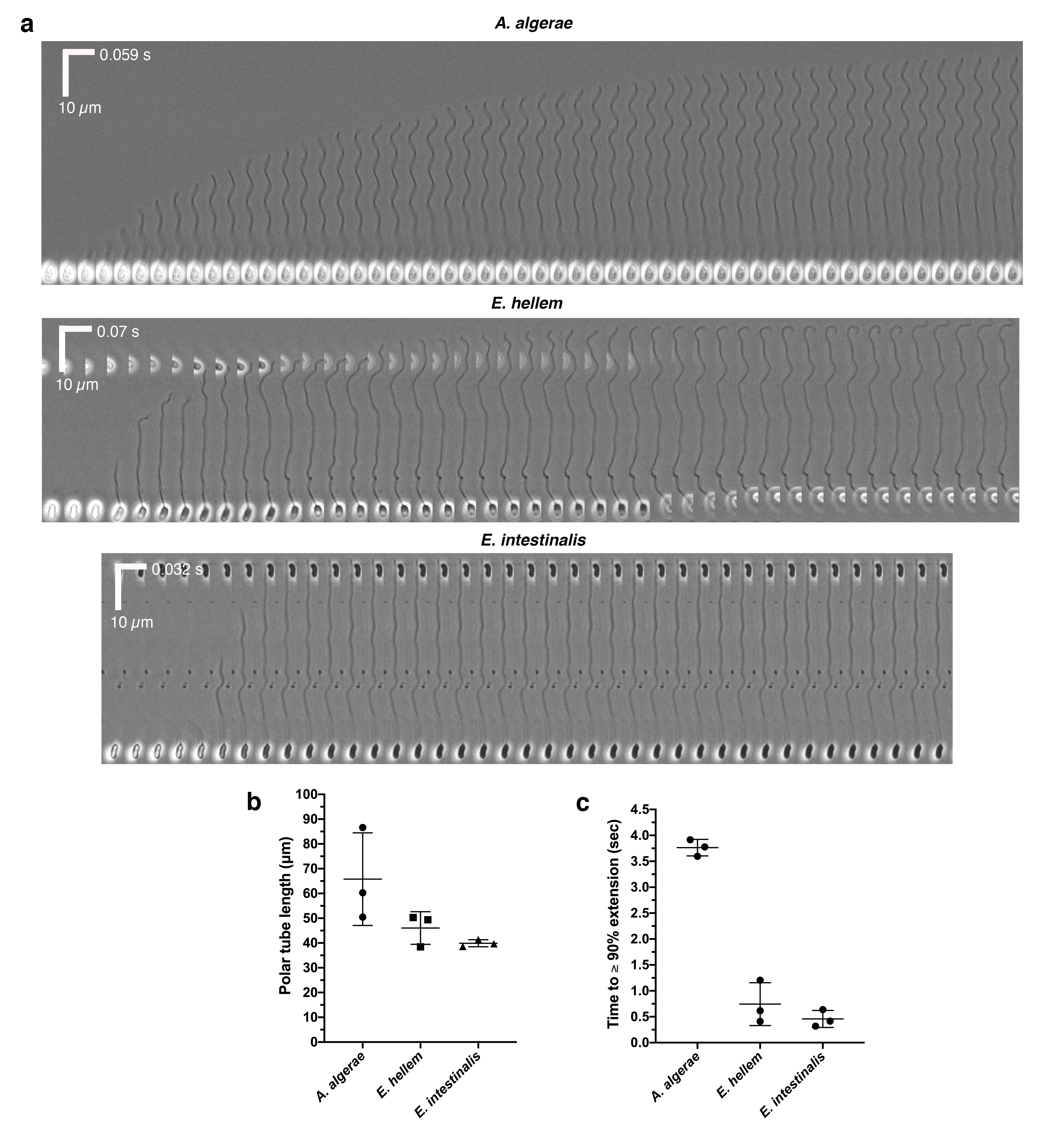

Supplement: S10 Fig — (a) Kymographs of incomplete spore germination from A. algerae, E. hellem, and E. intestinalis. Scale bar for time is shown on the X-axis and for distance on the Y-axis. For incomplete germination events observed, the distal end of the tube appeared to be straight, in contrast to the hooked ends usually observed for complete germination[25]. (b) PT length quantified from incomplete germination events. (C) Quantification of the time for the PT to reach ≥ 90% of its maximum length. The error bars in this figure represent standard deviation (n = 3 for each species). (TIF) [file ppat.1008738.s010.tif]

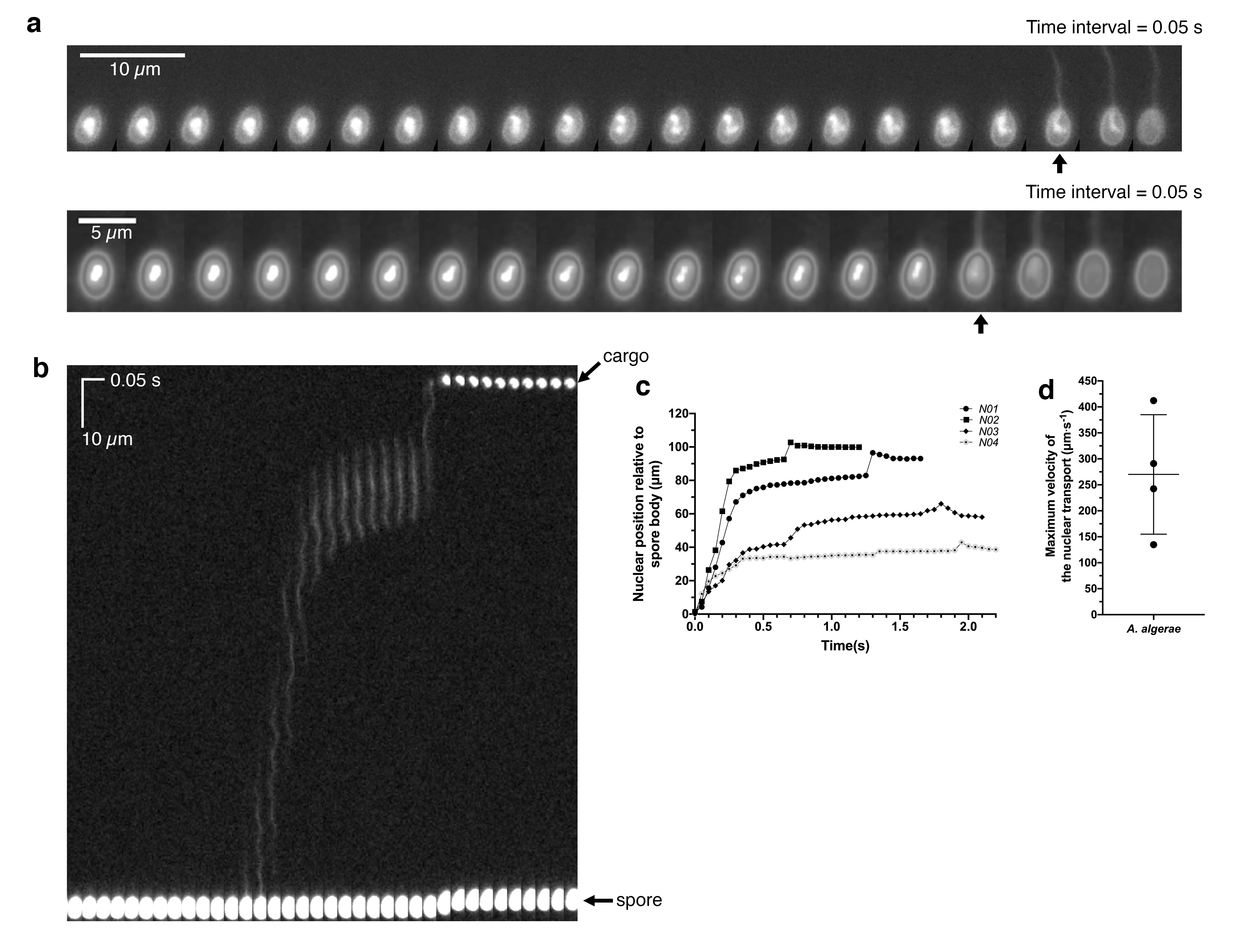

Supplement: S11 Fig — (a) Additional time-lapse images of fluorescently labeled nuclei inside spores during PT germination. Top and bottom represent two individual spores. Time intervals are 50 ms. Black arrows indicate the frame in which the nuclei have begun to enter the PT. (b) Additional kymograph of the nuclear translocation through the PT. (c) Quantification of the nuclear position relative to spore coat over time (n = 4). (d) Graph showing maximum velocity of the cargo transport process. The data was calculated from (c). Error bar represents standard deviation (n = 4). A total of seven movies were recorded to monitor nuclear transport. However, the nuclear signal in the first few frames of the movie was below our detection threshold for three of these movies, which have not been quantified here. (TIF) [file ppat.1008738.s011.tif]

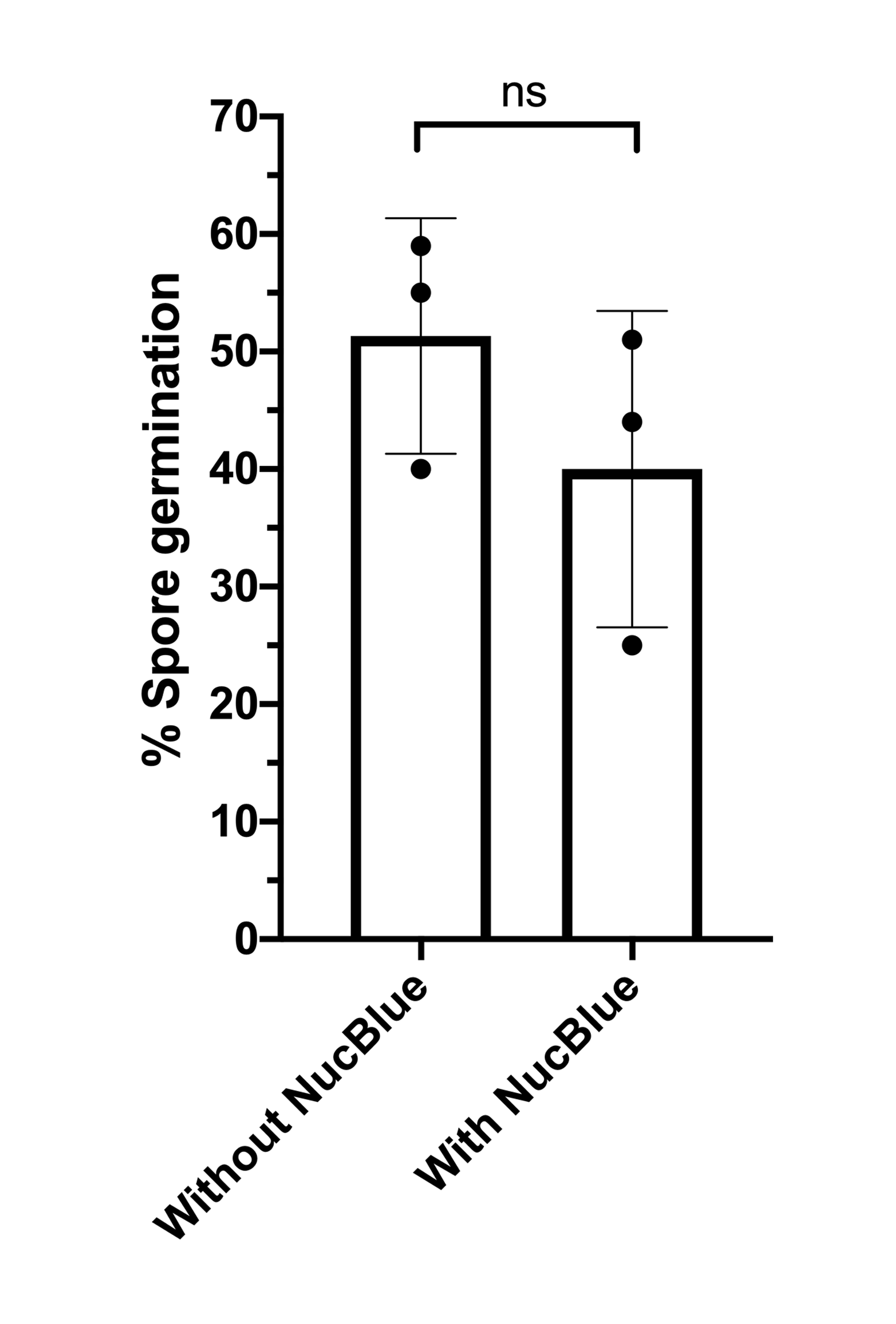

Supplement: S12 Fig — Graph indicates percent germination of spores in the presence or absence of NucBlue (3 independent preparations of A. algerae spores; ns = not statistically significant; p = 0.306, unpaired Student’s t-test). (TIF) [file ppat.1008738.s012.tif]
