# Supplementary material for: 3-Dimensional organization and dynamics of the microsporidian polar tube invasion machinery
Source: PLoS Pathog. 2020 Sep 18;16(9):e1008738. doi: 10.1371/journal.ppat.1008738 (PMC7526891; doi:10.1371/journal.ppat.1008738)
Supplement: Attachment — Submitted filename: 105558_1_rebuttal_1779721_qbvlsn.pdf [file ppat.1008738.s026.pdf]

Response to the reviewers:

We thank all three reviewers for their positive evaluation on our work. We appreciate the comments from the reviewers, which have helped us to improve our manuscript. Reviewer comments are in black and our responses are in blue. In the revised manuscript, changes are tracked in red. All page numbers refer to the revised manuscript.

Reviewer #1 (Evidence, reproducibility and clarity (Required)):

Summary

The authors use serial block-face scanning electron microscopy to reveal the architecture of two human-infecting microsporidia species. They describe in detail the structure and organisation of the polar tube invasion apparatus and other organelles. They capture spore germination and nuclei translocation in vitro using high speed microscopy. Analyses reveal the rapid kinetics of polar tube firing and show how the nucleus undergoes extreme deformation to travel through the narrow polar tube. This paper is well written, the figures are nicely executed, and the movies beautifully illustrate microsporidia biology. The authors also provide a sufficient level of detail for experiments to be reproduced and the data shown support the authors' conclusions and their model of polar tube-mediated invasion.

Major points

• Providing a supplemental table with all of the collective measurements of individual spores would be useful for looking at the correlation between different parameters such as the number of polar tube coils, spore size, angle with respect to the A-P axis, and if the ends are tangled.

We have now included Supplementary Table 1 providing all of the measurements of individual spores from SBFSEM experiments.

• The authors say that it is likely that the tangled ends of A. algerae may account for unsuccessful germination events. They also show that there are incomplete germination events, but do not comment on the frequency. It would be helpful to quantify in both A. algerae and E. hellem what percent of spores fired, what percent of spores had incomplete germination, and what percent of spores did not fire. It would also be useful to show if E. hellem ever has any tangled ends. This additional data would provide support for the authors conclusion.

This would indeed be interesting to follow up on. However, the caveat is that germination rates can vary quite considerably between different preparations, and also with the age of the spores. This is the reason we do not comment on it in the manuscript. The spores prepared for SBFSEM come from a different purification, and therefore, it would not be

informative to directly compare the germination frequency and the frequency of tangled ends. Tangled ends may lead to incomplete germination; however this is just one possibility. We have now included other possibilities for this observation (page 7).

For the second question, we did not observe tangled ends in *E. hellem* in our reconstructions. All PT ends are normal, and we note this in the legend of Fig. 3 (page 20) and in Supplementary Table 1.

Minor points

• No vacuole was observed for E. hellem and no anterior polaroplast was observed for A. algerae. Do the authors think these structures don't exist in these respective species or were they not detected by the block-face scanning EM?

A previous study by Bigliardi et. al, *Microbes and Infection*, 2001 (DOI: 10.1016/s1286-4579(01)01393-4) as well as our TEM study of germinated *E. hellem* showed that vacuole is present. We do not see it in SBFSEM, and it is also not clearly observed in TEM of dormant spores, possibly due to the smaller size.

The anterior polaroplast of *A. algerae*, is clearly observed by TEM analysis (Supplementary Fig. 4a). However, we do not observe this structure with SBFSEM, and therefore have not segmented it. This is now noted in the legend of Fig.1 (page 19).

• The right-handedness of the A. algerae helix is interesting. Does this observed directionality also apply to E. hellem?

We agree - this is one of our favorite observations! The right-handedness of the PT does also apply to all *E. hellem* spores as well. We apologize that this wasn't noted and have added this information into the main text (page 9) and Supplementary Table 1.

• In Figures 4F & S9, the way the polar tube shortening data are displayed make it difficulty to determine how often there is shrinkage after firing. The data should be represented differently, e.g. as a decrease in polar tube per individual spore, or % reduction per individual spore. Alternatively, the authors could pair each individual spore before and after shortening with connecting lines.

We have changed our graph to show the % reduction in length per spore as the reviewer suggested (Fig. 4g), and in addition show each individual spore before and after shortening in Supplementary Fig. 9b.

• In the discussion (page 9, line 5), the authors write 'the cargo regains its initial shape after exiting the PT'. The authors should either provide data on the shape of the nucleus before firing or reword this claim.

This is a good point. We have modified the wording in the main text, to indicate that the nucleus regains a globular shape (no longer deformed), rather than using the term "regains its initial shape" (page 15).

• NucBlue is used to visualise translocation of the nucleus through the polar tube. Can the authors comment or show data to indicate whether this stain affected normal germination processes?

We have quantified the effect of NucBlue on spore germination. The difference in percent germination from 3 batches of spores is not significantly different in the presence or absence of NucBlue. We have noted this in the methods section (page 34), and added the result to Supplementary Fig. 12.

• The polar tube being longer inside the spore than after firing has been previously observed and should be cited: Weidner E. Ultrastructural study of microsporidian invasion into cells. ZParasitenkd. 1972;40(3):227-42.

We thank Reviewer 1 for this suggestion, and have cited the paper (pages 11-12).

• Algerae polar filaments have been previously reported to shorten after reaching a maximum length and should be cited: Frixione, E. et al. Dynamics of polar filament discharge and sporoplasm expulsion by microsporidian spores. Cell Motility and the Cytoskeleton. 22, 38-50 (1992).

We have cited this paper (page 12).

• 'Fixed cell imaging' method subheading probably does not mean that and should be changed to 'Stained cell imaging'

We retained the subheading, as it was used to differentiate "live cell imaging" from non-live cell ("fixed cell") imaging. Both live and fixed imaging was done using fluorescent dyes as well as brightfield imaging.

• Figure 3C of E. hellem mentions a vacuole in the figure legend but is not shown. Please amend the figure legend.

We thank Reviewer 1 for pointing out this mistake. We have corrected the legend of Fig. 3.

• Figure 4, C-E. The lines indicating what is being statically compared are somewhat ambiguous. Using brackets would make it more clear which samples are being compared.

We have now changed the statistical comparison to brackets in all figures as suggested.

• Figure S1D measuring the angle of the polar tube should be referenced within the text.

We have referenced Supplementary Fig. 1D in the main text (page 7).

• Figures 4F and S9A: 'maximun' should be changed to 'maximum'.

We apologize for the typos. We have corrected them.

• Figure S8 is first referenced when describing how the polar tube emerges off-center in E. hellem and E. intestinalis (and later when discussing incomplete germination). To avoid confusion, the figure legend titled 'Examples of incomplete germination events' should be amended to also include the first observation.

We now refer to two separate figures for these two points, as we should not have used the incompletely germinated spores to make this point. We have added Supplementary Fig. 8 to show how the PT emerges off center.

Reviewer #1 (Significance (Required)):

Significance
Microsporidia have a unique invasion apparatus consisting of a polar tube that is required for the transfer of infectious cargo into host cells, as well as other poorly described organelles. Using scanning electron microscopy, the authors describe these organelles in the finest detail to date and show how the organelles of the dormant spores are arranged relative to one another. The authors also reveal the rapid germination kinetics of microsporidia in vitro using high speed microscopy. Microsporidia nuclei are much larger than the narrow polar tube, and the mechanism of nuclear transfer has remained mysterious for many years. In this paper, the authors use assays to show that the host nuclei undergo extreme nuclear deformation to aide passage through the polar tube. This is the first time this process has been visualised and represents a significant advance in our understanding of these parasites. Comparisons between the architecture and

germination kinetics of multiple microsporidia species add weight to the paper and reveal conserved and distinct features within the phylum.

The findings will be of broad interest to those interested in pathogen invasion mechanisms and more specifically to microsporidia researchers. Little is known about the infection biology of microsporidia, and this deep look into the dynamic infection apparatus adds significantly to the understanding of these microbes.

Our research group studies the natural microsporidia pathogen Nematocida parisii in C. elegans. We investigate host factors important for infection as well as immunity to N. parisii through the use of mutants and wild isolates. We characterize the microsporidia infection process in vivo using assays to determine host cell invasion

----

Reviewer #2 (Evidence, reproducibility and clarity (Required)):
This is a very nice paper that provides useful descriptive data on the process of germination of microsporidia and the organization of the polar tube. Despite this structure having been described over 125 years ago and being the subject of EM studies for over 50 years, our understanding of how this structure works during invasion is still quite limited. This paper provides excellent evidence for its structure in the spore and during germination. The paper is written clearly, the evidence is well presented and the presentation has excellent clarity. The conclusions are well supported by the data.

I do not think any additional experimentation is required and I would accept the paper and publish as written.

Reviewer #2 (Significance (Required)):
This provides a significant increase in data and is an advance in understanding the structure of this invasion apparatus as well as in the biophysical properties of this the polar tube.

The appropriate literature is cited and incorporated into the presentation of this data.

The text and figures were excellent and the supplemental videos are of significant value.

We thank Reviewer 2 for their positive comments on our work.

Cross-referenced comments from Reviewer #1 on Reviewer #3

Main comment 3:

Although more videos of the nuclei firing would be nice, this additional data is unlikely to change the conclusions.

We agree that this is the case, and thank Reviewer 1 for pointing it out. We indeed would have liked to have more movies; however this imaging is incredibly challenging and already represents several months of optimization.

Minor comment 5: 3d reconstructions of E. intestinalis would be cool, but not necessary unless the authors already have the data.

We agree it would be nice to have these data for many species and indeed that represents future work in our lab. SBFSEM is very time-consuming, and it is therefore not trivial to obtain 3D reconstructions for many species. We thank Reviewer 1 for commenting on this point.

Reviewer #3 (Evidence, reproducibility and clarity (Required)):

The article submitted by Jaroenlak et al is a very clear and carefully presented manuscript, focusing on microsporidian polar tube invasion machinery. Microsporidia belong to a phylum of small unicellular eukaryotes all obligate intracellular parasites which are able to form environmentally resistant spores. These fungi-like parasites can infect most animal groups and are of economic, veterinary and medical importance. Interestingly, Microsporidia possess an invasive apparatus composed of a highly specialized structure, the polar tube, that is unique in the eukaryotic world and represents one of the most sophisticated infection mechanisms. The invasion process involves the rapid extrusion of the spore polar tube that can penetrates the host cell membrane for the delivering of the spore content (sporoplasm) inside this cell. However, very little is known regarding both structure and functioning of the polar tube, mainly because of the lack of genetic approaches for these intracellular parasites.

In this paper, the authors described nicely microsporidian spore structure with the coiled polar tube (3-D organization) and polar tube discharge associated with germination kinetic using a variety of imaging methods (Serial block-face scanning electron microscopy and live-cell imaging of polar tube firing) to better understand this unique and fascinating invasion process.

The manuscript is well written and contains really nice 3-D pictures associated with videos and complete a recent paper published by Takvorian et al. (2020) on the polar tube architecture of the microsporidian species Anncaliia algerae. Their data give new insight

to decipher the devagination process that is not trivial because of the high velocity of the phenomenon (less than 2 sec for the whole process i.e. polar tube extrusion and sporoplasm release). Thanks to their observations, they proposed a 3D reconstruction of the spore with a detailed description of intrasporal organelles as a polar tube germination model mainly focusing on A. algerae model.

In my opinion, few points need to be improved

1.Most of the work was done on A. algerae (large spore size, medical importance, wide host range from invertebrates to vertebrates). The characteristics of this species should be more detailed in the introduction and also indicated in the abstract. The use of the other related species (Encephalitozoon hellem and E. intestinalis) is not clearly justified. The 2D/3D description for E. hellem needs to be more detailed: number of PT coils, orientation,... as for A. algerae to have a real comparison.

We have now incorporated the species names including "*A. algerae*" into the abstract as requested. *E. hellem* and *E. intestinalis* were used to assess differences and similarities in different species, as described on page 9 and in the kinetics section. We have also included medical relevance with appropriate references (page 4). We have now provided detailed information as requested for 3D reconstructions and PT kinetics for all species in Supplementary Table 1.

2.I suggest to add a paragraph about polar tube proteins (5 PTPs have been described in microsporidia) in the introduction and/or discussion sections. Indeed, polar tube length modifications may be also explained by the elasticity conferred by polar tube proteins. This could be discussed in the article.

The PTPs are indeed very interesting. We have added some information about PTPs in the main text as suggested (page 7). However, we think that adding a whole paragraph on PTPs is beyond the scope of this paper since this body of work does not shed light on the polar tube proteins per se.

3.In the last part of their manuscript, the authors focused on the cargo transport through the polar tube using nucleus tracking (NucBlue dye). This study was only performed in A algerae spores (probably due to the PT size). For the velocity measure as well for the nuclei deformation during their transit, they worked on a reduced number of samples (4 and 7, respectively). I think they could increase this number at least to ten to have more robust data. They clearly observed a huge nuclei deformation to fit through the narrow polar tube before getting back to their globular shape when they are released at the tip of the polar tube. The authors compared this phenomenon with nucleus deformation

observed during tumor cell invasion associated with DNA damage. I'm really septic concerning this part because division of parasites is highly efficient a few hours after host cell infection. Moreover, recent transcriptomic data on Nosema bombycis sporoplasm did not reveal any enzymes involved in DNA reparation (He et al., 2020).

We indeed would love to obtain more movies for the nuclear translocation. However, this experiment is extremely technically challenging, due to the low signal to noise, fast speeds, and unpredictability of PT germination. Given the stochastic nature of the experiment, acquiring additional data may take several weeks or months of effort, and as Reviewer 1 pointed out, obtaining more than n=7 movies will likely not change the conclusions.

We thank the reviewer for pointing us to the He, et al. 2020 reference. We have reviewed the Supplementary Table S2 from this paper and found that transcripts for many DNA repair enzymes are found in the *N. bombycis* sporoplasm. However, out comment about DNA repair in the sporoplasm was purely speculative, and may or may not be the case. This is not an essential point of our manuscript, so to avoid any confusion, we have removed this concept from the Discussion (page 16).

4. In the discussion section, recent references need to be included, including the recent paper on A. algerae PT description published by Takvorian et al (An ultrastructural study of the extruded polar tube of Anncaliia algerae (Microsporidia). 2020). The authors should compare their data with those of the Takvorian paper.

While we had previously cited this paper and compared our data (for example, in the legend of Fig. 4), we have now expanded our discussion and comparisons with Takvorian, et al, including in the introduction (page 4) and result sections (pages 7, 12). We have also cited the paper in the context of PT diameter measurement (page 13).

Minor comments :

1.M&M, for the nucleus dye NucBlue could you precise the concentration used rather than the volume

We contacted Thermo Fisher during the preparation of this manuscript to try to obtain detailed information about their NucBlue product. Unfortunately, their technical support responded that the NucBlue formulation is proprietary, and would not disclose the concentration in the stock solution. Thus, we are only able to provide the volume and catalog number of the NucBlue solution that we used.

2.M&M, section "germination conditions for microsporidian spores". Precise the germination conditions to get 70% germinated spores : incubation time, temperature...

We have added the detailed germination conditions used to obtain 70% germination in the methods section (page 31).

3.Could the abnormal tangled end of the PT be explained by the fact that these spores are not really mature as the culture of parasites is asynchrone?

We concur that different developmental stages of the spores could be an alternative explanation of the PT tangled end. We have added this possible explanation in the main text (page 7).

4.For E. hellem, the time to reach 90% PT extension seems to be highly variable (290 +- 200 ms) when compared to E. intestinalis 160 +- 20 ms) or A. algerae (830 +- 170 ms). Do you have any explanations for such variations?

This is an interesting observation, and at this time, we do not have a good explanation for the larger variation in *E. hellem*, but we do consistently observe more variation in *E. hellem* than other microsporidian species.

5.FigS9C : There is no 3D data for E. intestinalis data. It could be interesting to have these data to compare all 3 species for all aspects.

We agree that it would definitely be interesting to include the 3D reconstruction of *E. intestinalis* (and furthermore, other species). However, as Reviewer 1 mentioned, this experiment will not be trivial. We feel it is outside the scope of a revision, and we hope to study|more species using SBFSEM in the future.

Reviewer #3 (Significance (Required)):
Very little is known regarding both structure and functioning of the microsporidian polar tube. In this paper, the authors provided new data on the polar tube architecture(3-D organization)and germination process. They described the different steps of the invasion process (polar tube elongation, transit of the infectious sporoplasm and its emergence at the tip of the extruded polar tube)in different microsporidian species, Anncaliia algerae and 2 Encephalitozoon species. They confirmed the extreme velocity of both polar tube extrusion (elongation) and transit of the infectious sporoplasm.

These data were generated using serial block-face scanning electron microscopy and live-cell imaging techniques. They should discuss and compare their data with those recently published by Takvorian et al (J Euk Microbiol, 2020)

Audience : the reported findings are of interest for scientists working on invasion mechanisms used by intracellular parasites to penetrate their host cells. The polar tube is characterized by interesting biophysical properties (extreme stability and high elasticity) : this could be of interest for scientists developing new bioinspired nanomaterials.

Expertise : I have strong expertise on Microsporidia, originating from more than 20 years of research in this field. My team pioneered microsporidian genomics by fully sequencing the first genome of this fungus group that of Encephalitozoon cuniculi in collaboration with the Genoscope of Evry. Since then, we also acquired a strong expertise in host-parasite interactions by using transcriptomic and proteomic tools. The team also has a long lasting experience concerning microsporidian biology and the characterization of components from both the invasive apparatus (polar tube) and the spore wall in different microsporidian species.
