# Supplementary material for: 3-Dimensional organization and dynamics of the microsporidian polar tube invasion machinery
Source: PLoS Pathog. 2020 Sep 18;16(9):e1008738. doi: 10.1371/journal.ppat.1008738 (PMC7526891; doi:10.1371/journal.ppat.1008738)
Supplement: Peer review history

# Author comment: Author response to previous submission

**DOI:** 10.1371/journal.ppat.1008738.r001

19 Jun 2020

Attachment Submitted filename: 105558_1_rebuttal_1779721_qbvlsn.pdf Click here for additional data file.

# Editor report: Decision Letter 0

**DOI:** 10.1371/journal.ppat.1008738.r002

**Authors:** Vern B. Carruthers

23 Jun 2020

Dear Dr. Bhabha,

We are pleased to inform you that your manuscript '3-Dimensional Organization and Dynamics of the Microsporidian Polar Tube Invasion Machinery' has been provisionally accepted for publication in PLOS Pathogens.

Before your manuscript can be formally accepted you will need to complete some formatting changes, which you will receive in a follow up email. A member of our team will be in touch with a set of requests.

Please note that your manuscript will not be scheduled for publication until you have made the required changes, so a swift response is appreciated.

IMPORTANT: The editorial review process is now complete. PLOS will only permit corrections to spelling, formatting or significant scientific errors from this point onwards. Requests for major changes, or any which affect the scientific understanding of your work, will cause delays to the publication date of your manuscript.

Should you, your institution's press office or the journal office choose to press release your paper, you will automatically be opted out of early publication. We ask that you notify us now if you or your institution is planning to press release the article. All press must be co-ordinated with PLOS.

Thank you again for supporting Open Access publishing; we are looking forward to publishing your work in PLOS Pathogens.

Best regards,

Vern B. Carruthers

Section Editor

PLOS Pathogens

Vern Carruthers

Section Editor

PLOS Pathogens

Kasturi Haldar

Editor-in-Chief

PLOS Pathogens

​orcid.org/0000-0001-5065-158X

Michael Malim

Editor-in-Chief

PLOS Pathogens

orcid.org/0000-0002-7699-2064

***********************************************************

This study represents a very thorough and careful analysis of microsopridium spore structure and the kinetics of PT discharge and nuclear translocation. Although the expert reviewers do not note any findings in the work that fundamentally change the way the field understands microsporidium invasion, the study provides first of its kind detailed quantification of structural and kinetic aspects and notes some interesting distinctions between different microsporidia. As an outsider to the field, I find the most interesting and novel aspects of the work lie in the remarkably fast kinetics of nuclear translocation and that the nucleus doesn't begin to translocate until the PT is ~50% extended. Although the process of microsporidium infection of host cells is very different from that of other microbes, I think that a wide swath of microbiologist would find the study to be extremely interesting and insightful, if not only for gaining an appreciation of the incredible diversity of how infectious agents go about their business of initiating infection and in some cases causing disease.

Reviewer Comments (if any, and for reference):

# Editor report: Acceptance letter

**DOI:** 10.1371/journal.ppat.1008738.r003

**Authors:** Vern B. Carruthers

5 Aug 2020

Dear Dr. Bhabha,

We are delighted to inform you that your manuscript, "3-Dimensional Organization and Dynamics of the Microsporidian Polar Tube Invasion Machinery," has been formally accepted for publication in PLOS Pathogens.

We have now passed your article onto the PLOS Production Department who will complete the rest of the pre-publication process. All authors will receive a confirmation email upon publication.

The corresponding author will soon be receiving a typeset proof for review, to ensure errors have not been introduced during production. Please review the PDF proof of your manuscript carefully, as this is the last chance to correct any scientific or type-setting errors. Please note that major changes, or those which affect the scientific understanding of the work, will likely cause delays to the publication date of your manuscript. Note: Proofs for Front Matter articles (Pearls, Reviews, Opinions, etc...) are generated on a different schedule and may not be made available as quickly.

Soon after your final files are uploaded, the early version of your manuscript, if you opted to have an early version of your article, will be published online. The date of the early version will be your article's publication date. The final article will be published to the same URL, and all versions of the paper will be accessible to readers.

Thank you again for supporting open-access publishing; we are looking forward to publishing your work in PLOS Pathogens. 

Best regards,

Kasturi Haldar

Editor-in-Chief

PLOS Pathogens

​orcid.org/0000-0001-5065-158X

Michael Malim

Editor-in-Chief

PLOS Pathogens

orcid.org/0000-0002-7699-2064
